# What is associated with openness to integrated sport among school-aged youth with disabilities? A cross-sectional study from Hungary

**DOI:** 10.3389/fspor.2026.1922255

**Published:** 2026-10-19

**Authors:** Anetta Müller, Attila Lengyel, Katalin Mező, Éva Bácsné Bába, Nóra Laoues Czimbalmos, Peter Židek, Ferenc Mező

**Affiliations:** 1 Institute of Sports Economics and Management, Faculty of Economics and Business, University of Debrecen, Debrecen, Hungary; 2 Primary and Pre-School Education, Faculty of Education, Univerzita J Selyeho, Komárno, Slovakia; 3 Institute of Psychology, Faculty of Pedagogy, Eszterhazy Karoly Katolikus Egyetem, Eger, Hungary

**Keywords:** adolescents, disability, inclusion, integrated sport, latent profile analysis, sport participation

## Abstract

**Introduction:**

Integrated sport—defined here as people with and without disabilities sharing a sport setting—may support social connection and opportunities for participation, but integration does not by itself establish inclusion, and stated openness is not actual participation. This cross-sectional study examined factors associated with whether school-aged youth with disabilities in Hungary said they would like to take part in an integrated sports club.

**Methods:**

A survey of 1,298 youth with disabilities aged 8–18 was conducted with the support of a national disability-sport federation. The single-item outcome retained its three nominal response options—“yes,” “no,” and “not interested”—without assuming that the latter two captured active refusal and passive disengagement. Gaussian latent profile analysis summarised seven activity-limitation indicators; a categorical latent class model tested sensitivity to their ordinal measurement; and multinomial logistic regression assessed associations with limitation severity, impairment type, awareness, access, attitude, and school type.

**Results:**

Activity limitation formed a low-to-high severity gradient. Average posterior classification probabilities were .953, .902, and .938, and the categorical sensitivity model reproduced the ordered structure (87.6% identical ordered assignment; adjusted Rand index = .689). The full multinomial model had modest explanatory power (McFadden's *R*^2^ = .088). Neither continuous severity (LR *p* = .087) nor the profile-membership block (LR *p* = .097) significantly improved the full contextual model. However, two individual profile coefficients remained statistically significant in the “not interested”-vs.-“no” contrast. Liking sport was the strongest correlate, although it was conceptually proximal to the outcome; its exclusion reduced McFadden's *R*^2^ to .068 but did not make severity significant. Awareness and perceived access were also associated with the response categories, and impairment type was associated particularly with choosing “not interested” rather than “no.”

**Discussion:**

The findings identify correlates of a stated, unvalidated openness item, not causes of inclusion or participation. Their interpretation is limited by the cross-sectional convenience sample, single-item measures, modest model fit, and the absence of participant-level records of questionnaire mode or pictorial support.

## 1 Introduction

Regular physical activity confers broad physical, psychological, and social benefits on children and adolescents, however, young people with disabilities consistently engage in lower levels of physical activity than their peers without disabilities (1–6). Population studies repeatedly place this group below physical-activity guidelines and at elevated risk of obesity, lower cardiorespiratory fitness, and poorer mental health, with consequences that track into adulthood (7–9). The gap is not a minor disparity at the margins of public health; it is one of the more durable inequalities in youth physical activity, and closing it has become a recognised policy priority across education and health systems (10, 11).

Sport occupies a special place within this agenda. Beyond its fitness benefits, organised sport offers social connection, friendship, perceived competence, and a sense of belonging that are particularly consequential for young people whose opportunities for informal recreation are already constrained (12–14). Increasingly, policy encourages integrated sport—settings in which people with and without disabilities take part together (10, 15). Integrated sport is held to deliver a double dividend: participatory and developmental gains for young people with disabilities, and, through sustained intergroup contact, more favourable attitudes among those without (16–18). The Hungarian setting in which the present study was conducted reflects this trajectory, with national bodies actively promoting integrated and disability sport for school-aged youth (19, 20).

In this article, integration, inclusion, participation, and openness are not treated as synonyms. Integrated sport refers to people with and without disabilities sharing a sport setting. Inclusion refers to the quality of that setting—whether participants experience belonging and whether provision is adapted to varied needs—rather than physical co-presence alone. Participation refers to actual behavioural engagement. Openness, as measured here, is a respondent's stated answer about joining a described integrated club; it may precede participation but is neither participation itself nor evidence that a setting is inclusive (9, 15, 21).

The expectation that contact produces favourable attitudes rests on a long tradition in social psychology. Allport's (22) contact hypothesis holds that intergroup contact under favourable conditions—equal status, common goals, cooperation, and institutional support—reduces prejudice, and the framework has been applied widely to disability and to inclusive education (23, 24). In sport specifically, adults involved in integrated activities report somewhat less negative attitudes toward people with intellectual disability than non-involved comparison groups (25), and structured contact in physical education has been associated with more favourable attitudes toward including peers with disabilities (16, 18, 26). Yet the evidence is more equivocal than the simple contact narrative implies. Effects are often confined to particular attitude components, mere placement without genuine cooperative engagement may do little, and intention does not translate cleanly into behaviour (25). Reviews of inclusive physical education likewise caution that shared physical space is not the same as inclusion, and that the conditions Allport specified are frequently unmet in practice (21, 27).

If contact alone is an unreliable basis, attention turns to what actually shapes a young person's openness to taking part in integrated sport. Three broad classes of determinant recur across the participation literature. The first is awareness: young people and families can only engage with provision they know exists, and limited knowledge of suitable clubs and opportunities is among the barriers most consistently reported by children with disabilities and their parents (28–30). The second is access: the perceived availability of suitable, reachable provision, together with the transport, cost, and facility constraints that recur across systematic reviews as the dominant environmental barriers for this group (29). The third is attitude—here the young person's own disposition toward sport, including how much they like it and whether they feel capable of doing it. Enjoyment and perceived competence are among the most consistently reported personal facilitators of participation and are tightly bound up with the self-efficacy that sustains engagement over time (28, 29, 31, 32, 63).

This awareness–access–attitude grouping is not a free-standing scheme but a focused reading of a broader tradition. Within the framework of the International Classification of Functioning, Disability and Health (ICF), participation has been analysed through five key environmental conditions—the “five A's” of availability, accessibility, affordability, adaptability, and acceptability (9, 32, 33). Availability and accessibility map closely onto what we term access; affordability refers to financial feasibility; adaptability concerns whether provision can be modified to individual needs; and acceptability concerns whether provision is experienced as appropriate and welcoming (22, 28, 29, 32). Reading openness to integrated sport through this lens keeps the present study aligned with the inclusive-participation literature in physical education and sport (21) while concentrating attention on the correlates measured here. These variables are treated as correlates of stated openness rather than established determinants of actual participation.

These questions are especially salient in Central and Eastern Europe, where provision for disability and integrated sport is still maturing and where the environmental barriers identified internationally are keenly felt. Most evidence on inclusive sport and on the barriers to disability-related physical activity has been generated in Western European, North American, and other comparatively well-resourced sport systems, and its conclusions need not transfer directly to settings with different institutional histories and levels of provision (34). Across much of Central and Eastern Europe, dedicated disability-sport structures, inclusive school provision, club-level opportunities, and accessible transport remain unevenly distributed, so that the awareness and access constraints that are comparatively marginal elsewhere may here be more decisive (35). In Hungary specifically, the absence of suitable sports facilities near the place of residence and the lack of peers to train with have been reported as leading reasons for non-participation among people with disabilities (36–38), and stakeholders across the region point to persistent structural and funding gaps in inclusive provision (39, 40).

The present study is situated in this context. Data were collected with the support of a national federation that promotes disability and integrated sport for school-aged youth, providing an unusually large window onto a population that is otherwise hard to reach, and allowing the awareness, access, and attitude determinants to be examined at a scale rarely available in this field.

A further consideration is whether openness is associated less with these contextual and attitudinal factors than with the severity of a young person's disability-related limitations. It is intuitive to suppose that the more an impairment limits everyday functioning, the less open a young person will be to sport with peers. Yet impairment severity and impairment type are distinct: severity indexes how much functioning is constrained, whereas type may carry its own social and motivational meaning (28, 33, 41). Disentangling the two matters both conceptually and practically, because interventions aimed at severe limitation differ sharply from those aimed at awareness, access, or attitude (31, 32). Latent profile analysis offers a way to summarise multiple limitation indicators into descriptive subgroups, which can then be related to an outcome of interest (42, 43).

Against this background, the present study draws on a large cross-sectional survey of school-aged youth with disabilities in Hungary (*N* = 1,298) to examine factors associated with a single item on openness to integrated sport. We retain the item's three response categories—“yes,” “no,” and “not interested”—rather than collapsing them into a binary outcome. This preserves potentially informative response differences, but the categories are treated as nominal labels: no independent measure of prior consideration, response certainty, or reasons for responding establishes that “no” represents active refusal or that “not interested” represents passive disengagement. The study addresses three questions. First, what latent structure underlies young people's reported activity limitations, and does it form qualitatively distinct profiles or a severity gradient? Second, do these profiles, or a continuous measure of limitation severity, add explanatory value for the openness response? Third, how are awareness, access, attitude, impairment type, and school type associated with the three response categories once severity is taken into account? Because the design is cross-sectional, all three questions concern association rather than causal influence.

## 2 Materials and methods

### 2.1 Sample and recruitment

The study followed a cross-sectional design and targeted school-aged children and adolescents with disabilities educated in two settings: segregated special-education institutions and mainstream institutions offering inclusive education. Recruitment was carried out with the assistance of the National Federation of Disabled Students' Sports (FODISZ), whose involvement was central to reaching this hard-to-access population and to coordinating questionnaire administration across the country. Working through FODISZ and through institutional leaders, who in turn liaised with families, participants were drawn from three types of setting: special-education schools, integrated education programmes, and community-based disability organisations. To be eligible, respondents had to be between 8 and 18 years of age, have at least one diagnosed disability, and provide assent together with parental consent in the case of minors. Recruitment was non-probability based; no population sampling frame or denominator permitting calculation of a population response rate was available.

The procedure yielded 1,298 valid respondents (*M* age = 13.97 years, SD = 2.90, range 8–18), of whom 55.7% were boys and 44.3% were girls. Most attended segregated special-education institutions (69.4%), and the remainder mainstream, integrative institutions (30.6%). The most frequent impairment category was physical or motor impairment (52.4%), followed by multiple disabilities (15.3%), visual impairment (13.3%), intellectual disability (8.4%), autism spectrum disorder (4.2%), speech impairment (3.4%), learning disability (2.3%), and hearing impairment (0.7%). The present analysis used the full pool of valid respondents. Full sample characteristics are reported in Table 1.

**Table 1:** Sample characteristics, outcome distributions, and key predictors.

Variable	Category	*n* (%)/*M* (SD)
Age (years)	–	13.97 (2.90)
Sex	Girl	575 (44.3%)
	Boy	722 (55.7%)
School type	Special (segregated)	900 (69.4%)
	Mainstream (integrative)	397 (30.6%)
Impairment type	Physical/motor	680 (52.4%)
	Multiple	198 (15.3%)
	Visual	173 (13.3%)
	Intellectual	109 (8.4%)
	Autism spectrum	54 (4.2%)
	Speech	44 (3.4%)
	Learning	30 (2.3%)
	Hearing	9 (0.7%)
Openness to integrated sport	No	497 (38.3%)
	Yes	494 (38.1%)
	Not interested	306 (23.6%)
Sport outside PE	No	527 (40.6%)
	Yes	771 (59.4%)
Likes sport	No	323 (24.9%)
	Yes	974 (75.1%)
Feels capable of sport	No	166 (12.8%)
	Yes	1,131 (87.2%)
Knows a relevant club	No	501 (38.6%)
	Yes	541 (41.7%)
	Does not want club sport	255 (19.6%)
Knows integrated sport exists	No	930 (72.1%)
	Yes	360 (27.9%)
Integrated option in area	No	213 (16.4%)
	Yes	142 (10.9%)
	Don't know	942 (72.6%)

Total sample *N* = 1,298; valid *n* varies slightly by variable. Percentages are based on valid responses.

### 2.2 Ethical considerations

The study was conducted in accordance with the principles of the Declaration of Helsinki. The research plan was reviewed and approved by the Research Ethics Committee of the Faculty of Economics and Business, University of Debrecen (resolution No. GTK-KB 006/2026). The Committee determined that the plan complied with ethical requirements and raised no ethical objections. Participation was anonymous and voluntary, and informed consent was obtained from participants and, where required for minors, from their parents or legal guardians.

### 2.3 Data collection procedures

Data were gathered over a 2-week period in May 2026, after ethics approval had been granted, using both paper-based and online questionnaires, so that respondents and the institutions supporting them could choose the more suitable format. The online version was built following accessible-design principles and was compatible with screen readers; for the paper-based version, trained assistants were available on site. In school settings, the questionnaire was completed during homeroom or other scheduled sessions in small groups, with teachers, special-education teachers, and physical-education teachers guiding the process, supported by a dedicated teacher's guide. Respondents recruited through community organisations completed the questionnaire at the organisation's premises. To accommodate the diverse needs of the sample, respondents could take breaks, and support with reading or marking answers was provided where needed; for children with intellectual disabilities or complex communication needs, pictorial supports clarified the response options. Completion took approximately 25 min on average (range 15–40), and all returned questionnaires were checked for completeness before analysis.

### 2.4 Measures

The items used here were drawn from a broader project questionnaire on the sporting habits, needs, and opportunities of school-aged youth with disabilities. The present analysis used the original item wording and response categories. Unlike formally validated multi-item measures developed for related constructs (44), the activity-limitation and openness measures used here were project-specific. No separate pilot dataset, test–retest assessment, cognitive-interview record, or formal construct-validation study was available for the present analysis. Accordingly, the analyses are exploratory. Internal consistency and dimensionality were evaluated for the seven-item activity-limitation set in the present sample; the single-item openness outcome does not permit internal-consistency analysis.

#### 2.4.1 Activity-limitation indicators

Seven items captured the degree to which respondents felt their disability limited them across distinct life domains: getting around (mobility), making friends, everyday tasks, leisure, learning, sport, and social integration. Each item was rated on a five-point scale from 1 (does not limit me at all) to 5 (limits me completely). These seven items served as the indicators for the latent profile analysis. An eighth, ambiguously worded item from the same questionnaire block was excluded because its content could not be unambiguously verified. Before modelling, the measurement properties of the seven-item set were examined through internal consistency, item–rest correlations, the inter-item correlation structure, and the eigenvalue pattern of the correlation matrix, together with the Kaiser–Meyer–Olkin (KMO) measure of sampling adequacy and Bartlett's test of sphericity (45, 46).

#### 2.4.2 Outcome: openness to integrated sport

The focal outcome asked whether the respondent would like to take part in an integrated sports club where people with and without disabilities exercise together. Responses fell into three categories: *no* (38.3%), *yes* (38.1%), and *not interested* (23.6%). These options were retained as nominal categories. No separate item measured prior consideration, response certainty, or reasons for the response; the data therefore do not establish that “no” represents active refusal or that “not interested” represents passive disengagement.

#### 2.4.3 Secondary outcomes and covariates

Two further outcomes indexed actual sport behaviour: any sport outside compulsory physical education (no/yes) and, among those active, its frequency. These outcomes were used to examine whether the three openness responses differed in concurrent behaviour. Covariates were grouped into three conceptual blocks alongside demographic controls. Awareness comprised knowing that integrated sport exists and knowing a relevant sports club; access comprised the perceived availability of integrated sport in the respondent's area; attitude comprised liking sport and perceived capability. All focal awareness, access, and attitude measures were single items. Demographic controls were sex, age, impairment type, and school type. Coding directions for all categorical variables were verified item by item against the study codebook. Multi-category nominal variables were entered as dummy-coded indicators rather than as ordinal scores: impairment type (reference: physical/motor impairment, the largest group) and perceived local availability, which had three options—don't know, no, and yes (reference: don't know). The three binary items (knows integrated sport exists, likes sport, feels capable) were coded 0/1.

The club-awareness item had three response options: no, yes, and “does not want club-level sport.” Because the last option is conceptually proximal to the focal outcome—both index a disinclination toward organised club sport—its inclusion as a predictor risks partial criterion contamination. The main model therefore used a clean binary contrast, knowing a relevant club (yes vs. no), and excluded the “does not want club-level sport” response from this contrast by folding it into the reference. This affected 255 respondents (19.6% of the sample), whose responses were therefore not interpreted as a distinct category in the main model.

### 2.5 Statistical analysis

Analyses proceeded in four stages: (i) examining the measurement properties and latent structure of the seven activity-limitation indicators, including an ordinal-indicator sensitivity analysis; (ii) modelling the three-category openness response as a function of awareness, access, and attitude, with demographic controls; (iii) testing the sensitivity of the model to the conceptually proximal liking-sport and club-awareness predictors; and (iv) testing whether activity-limitation severity—expressed as a continuous score or as profile membership—added explanatory value, and relating the response categories and profiles to actual sport behaviour.

#### 2.5.1 Latent profile analysis

Latent profile analysis (LPA) was conducted in R with the tidyLPA package (47) using the mclust estimator (48). The analysis was exploratory in aim: it tested whether the seven limitation indicators formed qualitatively distinct, domain-specific profiles or instead discretised a single underlying severity continuum. Because the indicators were five-category ordinal variables, the Gaussian LPA was treated as an exploratory approximation. The LPA was not treated as preferable to a dimensional model; rather, it provided a descriptive discretisation that allowed the research question about profile membership to be tested alongside the continuous mean severity score. One- to six-profile solutions were estimated. Two parameterisations were specified *a priori*: an equal-variance, zero-covariance model (mclust “EEI”) and a freely varying-variance, zero-covariance model (mclust “VVI”). The varying-variance parameterisation did not yield an admissible solution for any model with two or more profiles; the estimator failed to converge, likely reflecting class-specific variances collapsing toward zero with discrete, bounded indicators. All reported Gaussian solutions therefore use the equal-variance parameterisation. Competing solutions were compared on the Bayesian information criterion (BIC) (49), the sample-size-adjusted BIC (SABIC) (50), the Akaike information criterion (AIC) (51), entropy (52), the smallest-class proportion, the bootstrap likelihood-ratio test (BLRT) (53), and substantive interpretability.

As a sensitivity analysis designed for categorical indicators, a conditional-independence latent class model treated each of the seven items as a five-category multinomial variable. One- to six-class models were estimated by maximum likelihood with an expectation–maximisation algorithm and 60 starts per model in Python 3.12 (NumPy 2.3.5, SciPy 1.17.0, scikit-learn 1.8.0). AIC, BIC, SABIC, entropy, and smallest-class size were inspected. For the three-class solution, expected item means, entropy, the percentage of identical ordered modal assignments, and the adjusted Rand index were compared with the Gaussian LPA.

#### 2.5.2 Predictors of openness to integrated sport

Openness to integrated sport was modelled with multinomial logistic regression, with “no” as the reference category so that the “yes”-vs.-“no” and “not interested”-vs.-“no” contrasts could be interpreted separately. The full model contained the awareness, access, and attitude indicators, the dummy-coded impairment categories, and the remaining demographic controls. Odds ratios are reported with 95% confidence intervals; the joint contribution of each predictor across both contrasts was assessed with Wald tests, and impairment type was tested as a single multi-degree-of-freedom block. Multicollinearity was screened with variance inflation factors (all below 1.6). Because liking sport is conceptually proximal to willingness to enter a sport setting, the full model was re-estimated without this predictor. This sensitivity analysis assessed both the reduction in overall fit and the stability of the other coefficients; it does not remove the common-method limitation of the remaining single-item predictors.

#### 2.5.3 Added value of severity and links to behaviour

To isolate the contribution of activity-limitation severity, four nested multinomial models were compared on the common *N* = 1,289 sample used in the original analysis: demographics only, including dummy-coded impairment type (Model A); demographics plus a continuous severity score, the mean of the available limitation indicators (Model B); demographics plus the awareness–access–attitude block (Model C); and the full model (Model D). Incremental fit was assessed with likelihood-ratio tests. Profile membership was then tested categorically on the *N* = 1,286 cases with complete profile assignments and complete regression data. Moderate- and high-limitation dummies, with the low-limitation profile as reference, were added first to the demographic model and then to the contextual model; each block test had four degrees of freedom because two profile coefficients were estimated for each outcome contrast. Average posterior membership probabilities were calculated within each modally assigned profile (62). Modal profile assignment was also related to the two behavioural outcomes; this remains a simplification relative to a full bias-adjusted three-step procedure (54, 55).

#### 2.5.4 Missing data

Missing data were minimal: no individual variable used in the models exceeded 0.6% missingness. The Gaussian LPA and categorical sensitivity analysis used 1,293 respondents with complete data on all seven limitation items. For the continuous severity score, the mean was calculated from available items: three regression cases contributed six of seven items, whereas a respondent missing all seven items was excluded. The main multinomial model therefore used 1,289 cases. The profile-dummy models additionally required a complete modal profile assignment and used 1,286 cases. Cases missing other model variables were excluded listwise.

## 3 Results

### 3.1 Sample characteristics

Table 1 summarises the analytic sample. Openness to integrated sport was distributed almost evenly across the three categories, and a majority reported some sport outside physical education (59.4%). Most respondents liked sport (75.1%) and felt capable of doing it (87.2%). Awareness of integrated provision was comparatively low: only 27.9% knew that integrated clubs exist, and only 10.9% were aware of integrated opportunities in their own area.

### 3.2 Measurement properties of the activity-limitation items

The seven activity-limitation items were suitable for dimensional analysis and showed good internal consistency (Cronbach's *α* = 0.851). The inter-item correlations were moderate and uniformly positive (mean *r* = 0.45, range 0.10–0.71), and sampling adequacy was high (KMO = 0.861), with Bartlett's test of sphericity confirming that the correlation matrix departed from independence [*χ*^2^(21) = 4,060.0, *p* < .001]. Item statistics are reported in Table 2. Item–rest correlations were acceptable to strong for most items but weaker for the learning item (*r* = 0.35) and, to a lesser degree, the sport item (*r* = 0.50); removing the learning item would have raised *α* only marginally (to 0.865), so all seven items were retained.

**Table 2:** Descriptive statistics and item–rest correlations for the seven activity-limitation items (*N* = 1,293).

Item	Mean	SD	Item–rest *r*	*α* if deleted
Getting around (mobility)	2.53	1.29	0.56	0.837
Making friends	2.72	1.41	0.73	0.812
Everyday tasks	2.44	1.30	0.75	0.811
Leisure	2.36	1.35	0.74	0.811
Learning	2.99	1.28	0.35	0.865
Sport	2.54	1.56	0.50	0.849
Social integration	2.80	1.39	0.67	0.821

Items rated 1 (does not limit me at all) to 5 (limits me completely). Item–rest *r* = corrected item–total correlation. Overall Cronbach's *α* = 0.851.

The eigenvalues of the correlation matrix were 3.81, 1.08, 0.64, 0.51, 0.40, 0.31, and 0.26. The first component accounted for 54.4% of the variance and dominated the remainder, with a pronounced drop after the first eigenvalue; although a second eigenvalue marginally exceeded unity, no interpretable second dimension emerged. The set is therefore best described as broadly unidimensional, capturing a single overarching severity of activity limitation. This supports both the aggregation of the items into a continuous severity score and their use as indicators in a latent profile analysis, while also anticipating the gradient structure reported below.

### 3.3 Latent structure of activity limitation

Fit indices for the one- to six-profile solutions are shown in Table 3. The BIC and SABIC continued to fall as profiles were added, and the BLRT was significant at every adjacent comparison; on statistical fit alone, no solution was unambiguously optimal. We retained the three-profile solution because solutions with four or more profiles subdivided the same severity continuum without producing a qualitatively new pattern and fragmented the sample, with the smallest class falling to roughly 11%. Entropy increased only marginally from .846 for three profiles to .856 for four profiles, then varied non-monotonically; this .010 difference was not treated as substantive evidence favouring four profiles. The three-profile solution was therefore retained as the most parsimonious interpretable discretisation of the broadly one-dimensional indicator set, not as a uniquely supported statistical optimum.

**Table 3:** Latent profile analysis fit indices (equal-variance parameterisation), one to six profiles.

Profiles	LogLik	AIC	BIC	SABIC	Entropy	Smallest class
1	−15,664.4	31,356.9	31,429.2	31,384.7	–	100.0%
2	−14,053.5	28,151.1	28,264.7	28,194.8	0.889	48.4%
3	−13,741.7	27,543.4	27,698.3	27,603.0	0.846	29.5%
4	−13,542.8	27,161.6	27,357.9	27,237.2	0.856	11.4%
5	−13,354.2	26,800.5	27,038.1	26,891.9	0.874	11.4%
6	−13,267.4	26,642.8	26,921.7	26,750.1	0.856	11.1%

The three profiles formed a severity gradient rather than a set of qualitatively distinct response patterns (Table 4). Profile 1 (“low limitation”; 39.3% of classified cases) scored low across all domains; Profile 2 (“moderate limitation”; 29.2%) scored uniformly in the middle range; and Profile 3 (“high limitation”; 31.5%) scored high throughout. Although the gradient dominated, a few domains deviated locally from their profile's overall level: learning was comparatively elevated in the low-limitation profile, and the social domains—making friends and integration—were comparatively elevated in the high-limitation profile. The overall pattern indicates that the profiles largely discretise a single underlying severity dimension, consistent with the high internal consistency of the indicators.

**Table 4:** Profile means on the seven activity-limitation indicators (equal-variance, three-profile solution).

Profile	Mobility	Friends	Daily	Leisure	Learning	Sport	Integr.	*n* (%)
1 Low	1.59	1.49	1.27	1.19	2.47	1.47	1.75	508 (39.3%)
2 Moderate	2.88	2.78	2.69	2.27	3.03	2.92	2.87	378 (29.2%)
3 High	3.36	4.21	3.69	3.92	3.60	3.52	4.07	407 (31.5%)

Cell entries are profile means on the 1–5 limitation scale. Daily, everyday tasks; Integr., social integration. Percentages are of the 1,293 classified cases.

Classification quality in the retained Gaussian solution was high: the average posterior probability of the assigned profile was .953 for the low-, .902 for the moderate-, and .938 for the high-limitation profile. The categorical latent class sensitivity analysis also produced three ordered low-to-high classes. It assigned 87.6% of respondents to the same ordered modal category as the Gaussian model (adjusted Rand index = .689), and its entropy was .858 compared with .846 for the Gaussian solution. BIC and SABIC in the categorical enumeration also continued to decrease through six classes, so this analysis supports the ordered gradient but does not make three classes a uniquely optimal statistical solution.

### 3.4 Profile composition and bivariate associations

Having established the severity gradient, we examined how the three profiles differed on demographic and outcome variables (Table 5). The profiles did not differ in age [*F*_(2, 1,290)_ = 1.98, *p* = .138] and differed only weakly by sex, with boys somewhat over-represented in the low-limitation profile [*χ*^2^(2) = 6.22, *p* = .045, Cramér's *V* = 0.069]. School type was related to profile membership [*χ*^2^(2) = 22.74, *p* < .001, *V* = 0.133]: young people in the high-limitation profile were more likely to attend integrative settings (39.3%) than those in the low-limitation profile (25.2%). This descriptive association does not identify a placement mechanism or show that school type caused profile membership.

**Table 5:** Composition of the three limitation profiles on demographic and outcome variables.

Characteristic	P1 Low (*n* = 508)	P2 Moderate (*n* = 378)	P3 High (*n* = 407)	Test
Age, M (SD)	14.17 (2.82)	13.83 (3.07)	13.86 (2.81)	*F* = 1.98, *p* = .138
Boys, %	59.4	51.1	55.3	*χ*^2^(2) = 6.22, *p* = .045
Integrative school, %	25.2	28.0	39.3	*χ*^2^(2) = 22.74, *p* < .001
Physical/motor impairment, %	64.2	47.6	41.8	*χ*^2^(14) = 171.1, *p* < .001
Multiple disability, %	3.1	18.8	27.3	(Same test)
Visual impairment, %	17.9	9.8	11.1	(Same test)
Intellectual disability, %	3.7	12.2	10.8	(Same test)
Autism spectrum disorder, %	2.8	5.8	4.4	(Same test)
Speech impairment, %	4.5	4.0	1.5	(Same test)
Learning disability, %	3.5	0.8	2.2	(Same test)
Hearing impairment, %	0.2	1.1	1.0	(Same test)
Sport outside PE (yes), %	61.4	58.2	58.5	*χ*^2^(2) = 1.22, *p* = .54
Openness: not interested, %	18.9	27.5	26.0	*χ*^2^(4) = 12.49, *p* = .014

Impairment-type rows show the complete eight-category distribution; the reported test refers to the full eight-category cross-tabulation. Percentages are column-wise within each profile (*n* = 1,293 classified cases).

Most strikingly, the gradient mapped onto impairment type [*χ*^2^(14) = 171.1, *p* < .001, *V* = 0.257]. The low-limitation profile was dominated by physical or motor (64.2%) and visual (17.9%) impairment, whereas multiple disabilities rose steeply across the gradient (3.1%, 18.8%, and 27.3% from the low to the high profile) and intellectual disability was concentrated in the moderate and high profiles. The severity gradient is therefore not impairment-neutral: it partly reflects which impairments most pervasively constrain everyday functioning, with sensory and physical impairments tending toward the lower end and multiple and intellectual disabilities toward the higher end. This composition is descriptive rather than deterministic—every profile contained a mix of impairment types—but it clarifies what the latent gradient is capturing.

Turning to the outcomes, the profiles were essentially unrelated to actual sport participation outside physical education [61.4%, 58.2%, and 58.5% active from the low to the high profile; *χ*^2^(2) = 1.22, *p* = .54]. At the bivariate level, the openness response differed modestly across profiles [*χ*^2^(4) = 12.49, *p* = .014, *V* = 0.069], driven mainly by a higher share of “not interested” responses in the moderate and high profiles (27.5% and 26.0%) than in the low profile (18.9%). The adjusted profile-block test reported below did not reach the .05 threshold, although two individual “not interested”-vs.-“no” profile contrasts remained statistically significant.

### 3.5 Predictors of openness to integrated sport

The full multinomial model was statistically reliable but explained a modest share of the outcome variation (McFadden's *R*^2^ = 0.088; likelihood-ratio test *p* < .001; *N* = 1,289); most heterogeneity in the response therefore remained unexplained. All variance inflation factors were below 1.6. Odds ratios with 95% confidence intervals are reported in Table 6. Because “no” is the reference outcome, an odds ratio above one indicates that the predictor raises the relative likelihood of “yes” or “not interested” rather than “no”; it does not establish a causal mechanism.

**Table 6:** Main multinomial model predicting openness to integrated sport (reference category: “no”; *N* = 1,289).

Predictor	OR (Yes)	95% CI (Yes)	OR (Not int.)	95% CI (Not int.)
Sex (boy)	1.23	[0.94, 1.61]	1.27	[0.92, 1.75]
Age	1.00	[0.95, 1.05]	0.97	[0.92, 1.02]
Impairment: Visual	0.62*	[0.42, 0.92]	0.60	[0.36, 1.00]
Impairment: Hearing^a^	4.04	[0.45, 35.91]	5.12	[0.48, 55.05]
Impairment: Speech	0.61	[0.29, 1.30]	1.23	[0.53, 2.85]
Impairment: Learning^a^	1.54	[0.55, 4.32]	6.01***	[2.12, 17.06]
Impairment: Intellectual	0.86	[0.51, 1.45]	2.10*	[1.19, 3.69]
Impairment: Multiple	1.08	[0.67, 1.73]	2.47**	[1.44, 4.23]
Impairment: Autism	1.02	[0.49, 2.13]	2.62*	[1.18, 5.85]
School type (integrative)	0.94	[0.68, 1.31]	0.64*	[0.42, 0.96]
Activity-limitation severity	1.06	[0.92, 1.23]	1.22*	[1.02, 1.45]
Knows integrated sport exists	1.41*	[1.01, 1.96]	1.59*	[1.09, 2.32]
Knows a relevant club (yes vs. no)	1.37*	[1.04, 1.81]	2.31***	[1.67, 3.21]
Area: no (vs. don't know)	2.73***	[1.83, 4.08]	2.81***	[1.77, 4.46]
Area: yes (vs. don't know)	0.93	[0.56, 1.52]	2.56***	[1.54, 4.25]
Likes sport	2.88***	[2.00, 4.15]	4.52***	[2.76, 7.42]
Feels capable of sport	0.65	[0.41, 1.02]	1.42	[0.73, 2.78]

a Estimates for hearing impairment (*n* = 9) and learning disability (*n* = 30) are based on small subgroups and have wide confidence intervals; interpret cautiously.*p < 0.05 (statistically significant at the 5% level).**p < 0.01 (statistically significant at the 1% level).***p < 0.001 (statistically significant at the 0.1% level).

Liking sport was the strongest individual correlate [joint Wald *χ*^2^(2) = 51.9, *p* < .001]. Relative to those who disliked sport, respondents who liked it were more likely to say “yes” than “no” (OR = 2.88, 95% CI [2.00, 4.15]) and also more likely to select “not interested” than “no” (OR = 4.52 [2.76, 7.42]). The raw distribution makes the direction concrete: “no” was the modal response among those who disliked sport (58.2%) but dropped to 31.7% among those who liked it. Because liking sport is conceptually close to willingness to enter a sport setting and was measured in the same questionnaire, these estimates should be read as proximal associations rather than evidence that liking sport is an independent explanatory mechanism.

Awareness and access were also associated with the response contrasts. Knowing a relevant club [*χ*^2^(2) = 25.4, *p* < .001] and awareness that integrated sport exists [*χ*^2^(2) = 6.7, *p* = .035] were associated with responses other than “no.” Perceived local provision showed a non-monotonic pattern: relative to “don't know,” respondents who judged that no local option existed were more likely to say “yes” than “no” (OR = 2.73 [1.83, 4.08]), whereas those who reported an available option were not more likely to say “yes” but were more likely to select “not interested” rather than “no” (OR = 2.56 [1.54, 4.25]). This pattern was obscured when local availability was treated as a single ordinal term and is recovered here through dummy coding.

Impairment type, entered as a categorical block, also contributed [*χ*^2^(14) = 42.8, *p* < .001], with the effect concentrated in the “not interested”-vs.-“no” contrast: relative to physical/motor impairment, respondents with learning (OR = 6.01), autism-spectrum (OR = 2.62), multiple (OR = 2.47), and intellectual (OR = 2.10) disabilities were more likely to select “not interested” than “no.” Estimates for the smallest categories—hearing (*n* = 9) and learning (*n* = 30)—had wide confidence intervals and should be interpreted cautiously. Activity-limitation severity showed a weak association in the “not interested” contrast (OR = 1.22 [1.02, 1.45]) but did not contribute jointly [*χ*^2^(2) = 4.88, *p* = .087]. Reintroducing the full three-category club-awareness item improved fit [LR *χ*^2^(2) = 48.69, *p* < .001], while the principal directions of the other predictors remained similar; because its “does not want club sport” category is conceptually proximal to the outcome, the cleaner binary version remains the main specification.

Removing the conceptually proximal liking-sport predictor reduced McFadden's *R*^2^ from .0876 to .0678. Continuous severity still did not improve the reduced model [LR *χ*^2^(2) = 3.32, *p* = .191], and the main awareness, access, and impairment-type directions remained similar. Perceived capability was notably specification-sensitive: without liking sport, its OR was 1.35 [0.93, 1.96], *p* = .118, for “yes” vs. “no,” and 4.03 [2.26, 7.16], *p* < .001, for “not interested” vs. “no.” The direction reversal from the main model's OR of 0.65 in the “yes” contrast cautions against a simple self-efficacy interpretation.

### 3.6 Added value of severity and links to actual behaviour

The continuous-severity comparison in Table 7 shows that severity added little to the nested models. Adding severity to the demographic model increased McFadden's *R*^2^ only from 0.0239 to 0.0242 and did not improve fit [Model A to Model B: LR *χ*^2^(2) = 0.70, *p* = .703]. By contrast, the awareness–access–attitude block raised *R*^2^ to 0.0859 [Model A to Model C: LR *χ*^2^(12) = 171.97, *p* < .001]. Adding continuous severity to the full specification produced no significant gain [Model C to Model D: LR *χ*^2^(2) = 4.89, *p* = .087]. The categorical profile test on the *N* = 1,286 common sample led to a similar but more qualified result (Table 8). Profile dummies did not improve the demographic model [LR *χ*^2^(4) = 3.73, *p* = .444] or the full contextual model at the .05 threshold [LR *χ*^2^(4) = 7.85, *p* = .097]. Within the latter model, however, the moderate (OR = 1.57 [1.06, 2.34], *p* = .025) and high profiles (OR = 1.55 [1.03, 2.33], *p* = .034) were each associated with selecting “not interested” rather than “no”; neither profile predicted the “yes”-vs.-“no” contrast. Thus the profile block provided no statistically significant overall increment, but the individual category contrasts preclude an unqualified claim that profile membership was unrelated to the outcome.

**Table 7:** Nested multinomial models predicting openness to integrated sport (common sample, *N* = 1,289).

Model	McFadden's *R*^2^	LR test vs. prior	*p*
A. Demographics (incl. impairment)	0.0239	–	–
B. A + severity	0.0242	*χ*^2^(2) = 0.70	0.704
C. Demographics + awareness/access/attitude	0.0859	*χ*^2^(12) = 172.0 (vs. A)	<.001
D. Full (C + severity)	0.0876	*χ*^2^(2) = 4.89 (vs. C)	0.087

Demographics comprise sex, age, dummy-coded impairment type, and school type. The awareness–access–attitude block comprises knowing integrated sport exists, knowing a relevant club (binary), perceived local availability (dummy-coded), liking sport, and feeling capable. Severity is the mean of the seven activity-limitation indicators.

**Table 8:** Nested multinomial models testing profile-membership dummies (common sample, *N* = 1,286).

Model	McFadden's *R*^2^	LR test vs. prior	*p*
Aₚ. Demographics	0.0237	–	–
Bₚ. Aₚ + profile dummies	0.0251	*χ*^2^(4) = 3.73	0.444
Cₚ. Demographics + awareness/access/attitude	0.0858	–	–
Dₚ. Cₚ + profile dummies	0.0886	*χ*^2^(4) = 7.85	0.097

Aₚ and Cₚ reproduce the demographic and contextual specifications on the profile-complete sample. Profile membership was represented by moderate- and high-limitation dummies, with low limitation as the reference. Each likelihood-ratio test has 4 degrees of freedom because two profile dummies were estimated for each of the two outcome contrasts.

Profile membership was unrelated to actual sport behaviour. Sport outside physical education did not differ across the three profiles [low 61.4%, moderate 58.2%, high 58.5%; *χ*^2^(2) = 1.22, *p* = .54], and among those who were active, out-of-school frequency did not differ by profile [*χ*^2^(6) = 5.31, *p* = .50]. The three openness responses, however, differed in concurrent participation outside physical education: 48.5% of respondents answering “no,” 61.5% answering “yes,” and 73.9% answering “not interested” reported such sport [*χ*^2^(2) = 52.00, *p* < .001, Cramér's *V* = .200]. Among active respondents, frequency did not differ across the three openness responses [*χ*^2^(6) = 7.09, *p* = .312, *V* = .068]. The “not interested” category therefore cannot be equated with general disengagement from sport.

## 4 Discussion

This study examined factors associated with a stated openness item among school-aged youth with disabilities. Three findings stand out. First, the seven activity-limitation indicators formed a low-to-high severity gradient rather than qualitatively distinct domain-specific profiles, and an ordinal-indicator sensitivity analysis reproduced this ordered structure. Second, neither the continuous severity score nor the profile block significantly improved the full contextual model, although two individual profile coefficients remained significant in the “not interested”-vs.-“no” contrast. Third, awareness, perceived access, liking sport, and impairment type were associated with the response categories, but the model's explanatory power was modest and liking sport was conceptually proximal to the outcome. These results concern correlates of a single stated response, not causes of inclusion or actual participation.

### 4.1 A severity gradient, not a sport-specific profile

The latent structure of activity limitation was graded. The three profiles differed mainly in overall level rather than shape, and no single life domain—including sport—defined a distinct subgroup. This is consistent with the high internal consistency and dominant first eigenvalue. The categorical latent class sensitivity model also produced an ordered low-to-high solution, with 87.6% identical ordered modal assignments and an adjusted Rand index of .689 relative to the Gaussian solution. The LPA should therefore be interpreted as a descriptive discretisation of a broadly dimensional construct, not as evidence of natural types or as analytically superior to the continuous score (56, 57). Its value here is limited but specific: it makes classification uncertainty visible and permits a direct test of the profile-membership research question.

### 4.2 Limited incremental contribution of severity

Across the continuous and categorical operationalisations, the severity measures did not provide a statistically significant overall increment once awareness, access, and attitude were in the model. For the profile block, this null result was not absolute: the moderate and high profiles were individually associated with “not interested” rather than “no,” although the four-degree-of-freedom block test was *p* = .097 and neither profile predicted “yes” rather than “no.” Profile membership was unrelated to actual sport behaviour. The evidence therefore suggests that severity contributed little at the model level, not that every profile–response contrast was zero. This pattern is consistent with literature emphasising environmental and motivational correlates alongside impairment-related constraints (29, 58), but the cross-sectional data cannot rank these factors causally.

### 4.3 Associations with awareness, access, and attitude

Awareness, perceived access, and sport-related attitude were associated with the response categories. Liking sport was the strongest single correlate, but it is also conceptually close to willingness to enter a sport setting and may partly reflect the same underlying disposition. Removing it reduced McFadden's *R*^2^ from .0876 to .0678, while continuous severity still did not improve the reduced model (LR *p* = .191). The awareness and access estimates remained directionally similar. Perceived capability was unstable across specifications: its “yes”-vs.-“no” OR changed from 0.65 to 1.35, and its “not interested”-vs.-“no” OR increased from 1.42 to 4.03 when liking sport was removed. This sensitivity cautions against interpreting the single-item attitude coefficients as independent mechanisms.

The access findings were not, however, monotonic. Relative to those who simply did not know what their area offered, young people who judged that no local option existed were among the most willing to take part—suggesting latent, unmet demand rather than disengagement—whereas those who reported an available option were more often “not interested.” Uncertainty, rather than perceived absence, accompanied refusal. This complicates any simple “build it and they will come” inference and points instead to the value of making provision both visible and known. Because the data are cross-sectional, these patterns describe associations rather than causal effects, and the direction of the availability–openness relationship cannot be settled here (58).

### 4.4 Response categories and the role of impairment type

Retaining all three response options was analytically useful because impairment type was associated primarily with choosing “not interested” rather than “no.” It does not follow, however, that the two labels measure passive disengagement and active, considered refusal. No item assessed prior deliberation, certainty, motives, or the social meaning of the response options. Concurrent behaviour points in the opposite direction from a general-disengagement interpretation: respondents selecting “not interested” had the highest rate of sport outside physical education. The observed impairment-type associations may reflect preferences specific to integrated club sport, wording or response-format effects, differences in comprehension or support during administration, or other unmeasured factors. Sensory load, predictability, social complexity, and fit with provision remain plausible hypotheses from prior literature (59–61, 65), but none was measured here and none can be presented as the mechanism producing the observed coefficients. The wide intervals for hearing and learning disability add further uncertainty. The study cannot determine whether “not interested” reflects indifference, uncertainty, lack of information, perceived irrelevance, a preference for other forms of sport, or other unmeasured factors.

### 4.5 Reconsidering the contact expectation

The school-type result also illustrates why integration and inclusion should not be conflated. Integrative schooling was not associated with saying “yes” rather than “no” (OR = 0.94 [0.68, 1.31]), but it was associated with a lower relative likelihood of “not interested” rather than “no” (OR = 0.64 [0.42, 0.96]). This isolated contrast does not show that mainstream placement increases openness. It is nevertheless consistent with the conceptual point that shared placement is only integration; inclusion additionally depends on belonging, adaptation, and the quality of participation (21, 22, 66). The study did not measure these school-level conditions, so the mechanism behind the contrast remains unknown.

### 4.6 Practical implications

The findings suggest several hypotheses for intervention rather than tested prescriptions. Awareness was associated with the response categories, which makes improved signposting through schools, disability organisations, and families a reasonable target for future evaluation. Respondents who believed no local option existed were relatively likely to say “yes,” a pattern compatible with unmet demand, but the cross-sectional design cannot show that providing or advertising an opportunity would change their response or lead to participation.

Positive, low-pressure experiences that build enjoyment and competence are likewise plausible targets (64) because liking sport was strongly associated with the outcome. However, the proximity of the liking-sport item to the openness item and the instability of perceived capability across specifications mean that these associations should not be treated as causal evidence. For respondents with cognitive, developmental, or multiple disabilities, the impairment-type results justify investigating whether predictable structure, graded competence demands, and sensory and social adaptations improve inclusion; they do not show that poor fit caused a “not interested” response in this sample. Future longitudinal or experimental studies should test whether making opportunities visible and adapting provision changes stated openness and actual participation.

## 5 Strengths

The study has several strengths. It draws on a large sample (*N* = 1,298) from a population that is difficult to reach and includes special, integrative, and community settings. Retaining the original three response options avoided discarding information through binary recoding, while the revised interpretation does not assign unvalidated psychological meanings to those options. The analysis dummy-coded nominal predictors, reported a continuous severity benchmark alongside the profiles, tested profile-membership dummies directly, quantified posterior classification quality, and checked the Gaussian profile structure with a categorical latent class model. Sensitivity analyses also examined the conceptually proximal liking-sport and club-awareness predictors.

## 6 Limitations

Several limitations qualify these conclusions. First, the study is cross-sectional and used non-probability recruitment through FODISZ, schools, and disability organisations. Youth connected to these networks may be over-represented, no population response rate could be calculated, and causal or population-representative claims are not warranted. Second, the focal outcome and the awareness, access, liking, and capability predictors were single self-report items from a project-specific questionnaire. The outcome categories have not been shown to distinguish active refusal from passive disengagement, and common-method and conceptual overlap may inflate some associations. The available data provided internal-consistency and dimensional evidence for the seven-item limitation set, but not test–retest, convergent, discriminant, or cognitive-interview evidence for the focal constructs. Third, questionnaire mode and the use of pictorial or other response support were not recorded as participant-level variables; their possible association with impairment type and response selection could not be tested. Fourth, the final model's McFadden *R*^2^ of .088 was modest, indicating that most outcome variation was not captured. Fifth, estimates for the smallest impairment categories, particularly hearing (*n* = 9) and learning disability (*n* = 30), were imprecise. Finally, the three-profile solution was selected for parsimony and interpretation rather than as a unique statistical optimum, and distal associations used modal assignment rather than a bias-adjusted three-step estimator. Average posterior probabilities exceeded .90 in all three profiles, but classification error was not eliminated.

## 7 Conclusion

Among school-aged youth with disabilities, a continuous limitation score and a three-profile severity gradient made little overall incremental contribution to a single-item measure of stated openness once the measured awareness, access, and attitude variables were included. Awareness, perceived local availability, liking sport, and impairment type were associated with the response categories, but the model explained a modest share of variation and several predictors were conceptually proximal to the outcome. The “no” and “not interested” options should be reported as nominal response labels rather than validated forms of active refusal and passive disengagement. These cross-sectional findings identify hypotheses about visibility, access, and the design of integrated provision; longitudinal and intervention research using validated measures is required to determine whether changing these conditions affects openness, inclusion, or actual participation.

## Data Availability

The original contributions presented in the study are included in the article/Supplementary Material, further inquiries can be directed to the corresponding author.
